# Orthorexia Nervosa: differences between clinical and non-clinical samples

**DOI:** 10.1186/s12888-021-03348-2

**Published:** 2021-07-08

**Authors:** C. Novara, E. Maggio, S. Piasentin, S. Pardini, S. Mattioli

**Affiliations:** Department of General Psychology, University of Padova, Via Venezia 8, 35131 Padova, Italy

**Keywords:** Orthorexia Nervosa, Eating disorders, Obesity, Dieting

## Abstract

**Background:**

Orthorexia Nervosa (ON) is a construct characterized by behaviors, emotions, and beliefs on eating healthy food and excessive attention to diet; moreover, dieting has been considered a risk factor in ON symptoms development. The principal aim of this study was to investigate the differences in clinical and non-clinical groups most at risk of ON. Aspects that could be associated with ON (Eating Disorders [EDs], obsessive-compulsive symptomatology, perfectionistic traits, anxiety, depression, Body Mass Index [BMI]) were investigated in all groups.

**Methods:**

The sample consisted of 329 adults belonging to four different groups. Three were on a diet: Anorexia/Bulimia Nervosa group (*N* = 90), Obesity/Binge Eating Disorder group (*N* = 54), Diet group (*N* = 91). The Control group consisted of people who were not following a diet (*N* = 94). Participants completed several self-administered questionnaires (EHQ-21, EDI-3, OCI-R, MPS, BAI, BDI-II) to assess ON-related features in different groups.

**Results:**

Analyses highlighted higher orthorexic tendencies in Anorexia/Bulimia Nervosa, Obesity/BED, and Diet groups than in the Control group. Moreover, results have shown that in the AN/BN group, eating disorders symptomatology and a lower BMI were related to ON and that in Obesity/Binge Eating Disorder and Diet groups, perfectionism traits are associated with ON.

**Conclusion:**

Individuals who pursue a diet share some similarities with those who have an eating disorder regarding emotions, behaviors, and problems associated with orthorexic tendencies. Moreover, perfectionistic traits seem to predispose to higher ON tendencies. In general, these results confirm the ON as an aspect of the main eating disorders category.

**Supplementary Information:**

The online version contains supplementary material available at 10.1186/s12888-021-03348-2.

## Background

The term Orthorexia Nervosa (ON) was coined by Steven Bratman [1], and it refers to an excessive attention on diet and a fixation on eating healthy food that could be associated with unhealthy obsessions [2]. ON is a multifactorial construct characterized by behavioral, cognitive, and emotional aspects; it often begins to prevent or manage an illness or any other factor that could be harmful to personal health [3]. ON could interfere with everyday life, and health could be compromised by nutritional deficiencies and malnutrition [3–5]. However, ON is not recognized as a nosological category by the Diagnostic and Statistical Manual of Mental Disorders-5 (DSM-5) [6], since it is not yet clear if ON could be considered as an independent mental disorder or a pathological eating habit [7]. ON shows relationships with the symptomatology associated with Obsessive-Compulsive Disorder (OCD) [8–10]; moreover, a relationship between ON and Eating Disorders (EDs) is widely documented [10–19].

ON seems related to maladaptive perfectionism [2, 10, 17, 20, 21] and anxiety and depression-related symptoms [22, 23]. Diet could be considered a risk factor for ON, although it is not easy to understand if the focus on food is due to a healthy diet, usually followed to lose weight, avoid medical problems [3], or an orthorexic behavior [24]. Literature has shown a relationship between dietary and restrictive behavior [25]; in particular, a restrictive diet may increase the risk for EDs [26, 27]. Moreover, it is important to investigate if certain dietary habits (i.e., vegan/vegetarian diet or excessive adherence to healthy eating habits) are chosen in order to legitimize avoidance towards food, especially in subjects with Anorexia Nervosa (AN) or Bulimia Nervosa (BN) [13, 28] and in subjects at risk for them [29]. Following a vegetarian/vegan diet could be considered a risk factor for ON [24, 30–32].

To the best of our knowledge, only one study [24] investigated the role of diet on ON. The authors highlighted that people who were following a diet and who were changing many of their eating habits had more orthorexic tendencies than those who did not follow a diet, compared to those who were following a less restrictive one: orthorexic tendencies might increase when a more restrictive food choice is adopted.

Therefore, diet could represent a risk factor for ON, but it has not been sufficiently investigated. Most studies on non-clinical at-risk populations found more orthorexic tendencies in health professionals such as doctors or dieticians [33–35], in students attending medical or nutrition/dieting faculties [36–38], in people who regularly practice fitness [39], and in performing artists [40]. However, no study investigated the role of diet on ON in clinical populations that follow a diet inside a rehabilitation program. Many AN and BN factors-related [41] (i.e., excessive focus on food, rigid adherence to diet, and food restriction) could also be involved in ON [16] and maintains the dysfunctional food pattern [19]. Patients with AN/BN diagnosis show high orthorexic tendencies and share some nuclear aspects with the ON-like intrusive thoughts about food, perfectionism, and low insight level [10, 13–15, 17–19, 42, 43]. For these reasons, patients with AN/BN could be considered populations at risk of ON [13].

Obesity is a medical condition not included in EDs [6], but it could be linked to them with other factors, such as internalized weight stigma and fear of fat [44]; unhealthy weight control behaviors could increase psychological and medical repercussions due to their high weight. For these reasons, during their lifetime, people with obesity often follow a diet for long periods. Body Mass Index (BMI) itself does not seem to correlate with ON [13, 40, 45–49]; however, a higher BMI, along with eating problems and obsessive-compulsive-like behaviors, can be associated with higher orthorexic tendencies [50]. Some studies have shown that as the BMI increases, so do orthorexic tendencies [2, 35, 37, 51]. People with higher levels of body fat, and therefore in a condition of overweight or obesity, could follow a diet to decrease weight and improve their physical health, risking increasing orthorexic tendencies [48]. Several studies have investigated ON in AN/BN or obesity populations [2, 11–19, 24, 30, 35, 37, 51, 52] but it was not investigated the differences in these clinical populations at risk of ON.

Therefore, it is important to understand how following a diet could expose people with EDs or obesity to a higher risk of ON. The principal aim of our study was to investigate the differences in two clinical samples composed of individuals with AN/BN and Obesity/Binge Eating Disorder (BED), representing categories most at risk of ON symptoms. Both groups were following a diet due to EDs or obesity (for health or weight loss reasons); therefore, they could be exposed to a greater risk of developing ON. The two groups were compared with a non-clinical group composed of people who voluntarily followed a diet and a control group who did not follow any diet. We expected that clinical groups (AN/BN, Obesity/BED) showed more ON tendencies and more characteristics related to ON, such as perfectionistic traits, EDs, OCD, depressive and anxiety symptoms, compared to the control group. Moreover, orthorexic tendencies were expected to be higher in people on a diet than in the control group. Finally, in the current study, we explored which of these variables are more related to orthorexic symptoms.

## Methods

### Participants

The sample consisted of 329 adult subjects divided into four groups: AN/BN, Obesity/BED, Diet, and Control (Table 1). The AN/BN group consisted of 90 patients consecutively recruited into the study as they were hospitalized in Eating Disorder Treatment Centers in Central and Northern Italy. According to DSM-5, all patients had received a diagnosis of AN or BN by clinicians.

**Table 1:** Sociodemographic characteristics

	AN/BN (*n* = 90) M (SD) 1	Obesity/BED (*n* = 54) M (SD) 2	Diet (*n* = 91) M (SD) 3	Control (*n* = 94) M (SD) 4	F or CHI and sign (p)	Partial η^2^	Bonferroni post-hoc comparison (p)
Age (years)	27.52 (10.58)	53.78 (14.47)	45.89 (12.71)	24.60 (5.08)	126.23_(3)_ (<.001)	.54	**2 > 1;3;4 (<.001)** **3 > 1;4 (<.001)**
School Years	14.61 (3.17)	13.09 (3.70)	14.23 (3.27)	16.72 (2.33)	18.67_(3)_ (<.001)	.15	**1 > 2 (<.05)** **4 > 1;2;3 (<.001)**
BMI (SD)	18.16 (5.09)	38.86 (7.31)	24.78 (5.53)	21.04 (3.17)	194.55_(3)_ (<.001)	.65	**2 > 1;3;4 (<.001)** **3 > 1;4 (<.001)** **4 > 1 (<.001)**
Gender Female n (%)	89 (98.9%)	37 (68.5%)	48 (52.7%)	90 (95.7%)	82.06_(3)_ (<.001)	–	–
Diagnosis	AN n(%) = 69 (76.67%) BN n (%) = 21 (23.33%)	Obesity n (%) = 31 (57.41%) Obesity+BED n (%) = 23 (42.59%)	NA	NA	–	–	–
Comorbidity Yes n (%)	28 (30.77%)	28 (51.85%)	NA	NA	–	–	–
Psychotropic Drug Yes n (%)	62 (69.23%)	29 (53.7%)	3 (3.19%)	NA	–	–	–

*AN* Anorexia Nervosa, *BN* Bulimia Nervosa, *BED* Binge Eating Disorder, *BMI* Body Mass Index, *SD* Standard Deviation

The Obesity/BED group consisted of 54 patients with obesity, admitted consecutively in nutritional rehabilitation departments in Central e Northern Italy hospitals to lose weight and improve their health. All patients received an obesity diagnosis, and based on the DSM-5, some of them also had a BED diagnosis.

In AN/BN and Obesity/BED groups, the diet was a part of the rehabilitation program.

The Diet group consisted of 91 patients admitted consecutively who were following the “zone diet” by personal choice with a dietician in northern Italy at the time of the administration. The questionnaires were either sent home by the dietician’s patients or were personally delivered by the doctor. Subsequently, they were filled out at home and returned to the dietician.

The Control group consisted of 94 selected students at the University of Padova in northern Italy who voluntarily participated in orthorexia research. Only participants who were not on a diet at the moment of enrollment were selected for this group. No participants had a diagnosis of a mental disorder or were taking psychotropic drugs.

The research was presented to participants in the different settings mentioned above. Participants were fully informed about the research aim, and those who voluntarily decided to participate gave their written consent and filled out all self-report questionnaires. The anonymity and the confidentiality of the collected data were guaranteed. This study was conducted according to the Declaration of Helsinki and was approved by the Department of General Psychology’ Ethical Committee, University of Padova.

### Measures

Participants of each group were asked to complete a demographic schedule to collect data about gender, age, years of education, weight and height from which BMI was calculated.

Furthermore, participants had to report if they had ever suffered or were suffering from a medical or mental disorder, if they were taking any psychotropic drugs and following any diet.

Moreover, clinicians filled out a clinical schedule in which they were asked to indicate BMI, the current psychological diagnosis, comorbidities, current drug treatments, and other medical disorders about individuals from clinical groups. Any severe psychiatric and neurological diseases were considered exclusion criteria in our research.

The following questionnaires have been administered.

The Eating Habits Questionnaire-21 (EHQ-21 [53]; the Italian version by [22]) is a 21-item self-report questionnaire investigating thoughts, emotions, concerns, and behaviors related to ON. Items are scored on a four-point Likert scale (“False, Not at All True”, “Slightly True” “Mainly True” and “Very True”). It is composed of three subscales: “Knowledge” assesses knowledge and behaviors, “Feelings” assesses positive emotions, and “Problems” subscale concerns problems related to rigid and healthy eating. Both the original and Italian validation of the EHQ show good internal consistency (0.82 < Cronbach’s α < 0.90) and test-retest reliability (0.50 < r < 0.81). For each group, the current study showed good internal consistency for the total score and the three subscales score, except for the “Feelings” subscale in the AN/BN group and in the Diet group, where it was acceptable (Table 2).

**Table 2:** Comparisons between the AN/BN, Obesity/BED, Diet and Control groups

	AN/BN (1) *N* = 90	Obesity/BED (2) *N* = 54	DIET (3) *N* = 91	CONTROL (4) *N* = 94	F _(d.f.)_	*p*	Partial η^2^	Bonferroni post-hoc comparison (p)	α
**EHQ-21-TOT**	55.27 (15.45)	47.91 (10.96)	50.99 (8.84)	36.22 (7.54)	50.19_(3)_	<.001	.32	1 > 2;4 (<.001) 4 < 2;3 (<.001)	(1) = .93 (2) = .88 (3) = .85 (4) = .85
**EHQ-21-PROBLEMS MEAN (SD)**	30.92 (10.23)	22.55 (6.01)	22.69 (5.86)	15.26 (3.50)	80.00_(3)_	<.001	.42	1 > 2;3;4 (<.001) 4 < 2;3 (<.001)	(1) = .92 (2) = .75 (3) = .81 (4) = .75
**EHQ-21-KNOWLEDGEMEAN (SD)**	13.33 (3.90)	13.94 (3.55)	16.38 (2.61)	11.86 (3.03)	30.44_(3)_	<.001	.22	4 < 1;2 (<.05) 3 > 1;2;4 (<.001)	(1) = .79 (2) = .75 (3) = .74 (4) = .79
**EHQ-21-FEELINGS MEAN (SD)**	11.01 (3.08)	11.42 (2.82)	11.91 (2.52)	9.11 (2.70)	17.49_(3)_	<.001	.14	4 < 1;2;3 (<.001)	(1) = .61 (2) = .72 (3) = .65 (4) = .71
**EDI-3-TOT MEAN (SD)**	179.84 (55.15)	121.89 (60.53)	57.97 (30.62)	62.18 (36.62)	143.64_(3)_	<.001	.57	1 > 2;3;4 (<.001) 2 > 3;4 (<.001)	(1) = .96 (2) = .97 (3) = .94 (4) = .96
**EDI-3-DRIVE FOR THINNESS MEAN (SD)**	17.99 (7.34)	12.98 (6.95)	5.92 (3.25)	4.12 (4.21)	119.72_(3)_	<.001	.53	1 > 2;3;4 (<.001) 2 > 3;4 (<.001)	(1) = .85 (2) = .79 (3) = .72 (4) = .82
**EDI-3-BULIMIA MEAN (SD)**	10.28 (10.17)	9.57 (8.14)	2.65 (3.56)	2.86 (3.20)	31.52_(3)_	<.001	.23	1 > 3;4 (<.001) 2 > 3;4 (<.001)	(1) = .93 (2) = .90 (3) = .84 (4) = .77
**EDI-3-BODY DISSATISFACTION MEAN (SD)**	25.93 (9.94)	24.08 (9.34)	10.73 (8.42)	12.18 (9.15)	61.00_(3)_	<.001	.36	1 > 3;4 (<.001) 2 > 3;4 (<.001)	(1) = .87 (2) = .81 (3) = .87 (4) = .91
**MPS-TOT** **MEAN (SD)**	113.94 (23.73)	93.32 (22.61)	94.43 (24.99)	95.53 (24.18)	15.27_(3)_	<.001	.12	1 > 2;3;4 (<.001)	(1) = .94 (2) = .92 (3) = .95 (4) = .95
**OCI-R-TOT MEAN (SD)**	25.77 (14.96)	13.53 (13.74)	13.55 (11.87)	8.55 (9.52)	30.96_(3)_	<.001	.22	1 > 2;3;4 (<.001) 3 > 4 (<.05)	(1) = .90 (2) = .94 (3) = .91 (4) = .91
**BAI-TOT MEAN (SD)**	26.67 (14.46)	9.36 (10.45)	7.36 (9.62)	9.50 (7.80)	60.50_(3)_	<.001	.36	1 > 2;3;4 (<.001)	(1) = .93 (2) = .93 (3) = .94 (4) = .88
**BDI-II-TOT MEAN (SD)**	30.14 (14.49)	11.87 (11.44)	6.88 (7.49)	7.10 (7.81)	97.75_(3)_	<.001	.48	1 > 2;3;4 (<.001) 2 > 3 (<.05)	(1) = .93 (2) = .94 (3) = .90 (4) = .92

*BMI* Body Mass Index, *EHQ-21* Eating Habits Questionnaire-21, *BAI* Beck Anxiety Inventory, *BDI-II* Beck Depression Inventory-II, *OCI-R* Obsessive Compulsive Inventory-*Revised, MPS* Multidimensional Perfectionism Scale, *EDI-3* Eating Disorder Inventory-3, *AN/BN* AnorexiaNervosa/BulimiaNervosa, *Obesity/BED* Obesity/Binge Eating Disorder, *SD* Standard Deviation, *n.s.* Not statistically significant, d.f. degrees of freedom, *α* Cronbach’s Alpha coefficient

The Eating Disorder Inventory-3 (EDI-3 [54]; the Italian version by [55]) is a 91-item self-report questionnaire investigating symptoms of Eating Disorders and related psychological features on a six-point Likert scale (“Always”, “Usually”, “Often”, “Sometimes”, “Rarely”, and “Never”). The EDI-3 is composed of 12 subscales: “Drive for Thinness”, “Bulimia” and “Body Dissatisfaction” assess symptoms of EDs, while “Low Self-Esteem”, “Personal Alienation”, “Interpersonal Insecurity”, “Interpersonal Alienation”, “Interoceptive Deficits”, “Emotional Dysregulation”, “Perfectionism”, “Asceticism”, “Emotional Dysregulation”, “Perfectionism” and “Maturity Fears” assess some psychological features considered as risk factors for an EDs. In the validation sample, consisted of patients with EDs, the EDI-3 internal consistency (0.90 < Cronbach’s α < 0.97) and test-retest reliability (*r* = 0.98) were excellent. Moreover, good internal consistency was highlighted in the Italian version (0.72 < Cronbach’s α < 0.94). On the current study, Cronbach’s α was good for the total score and its subscales (Table 2). In the “Perfectionism” subscale, Cronbach’s α was acceptable for the AN/BN, Obesity/BED, and Diet groups. For the “Asceticism” subscale, Cronbach’s α was not acceptable, so we did not consider it in comparisons between groups.

The Obsessive Compulsive Inventory-Revised (OCI-R [56]; the Italian version by [57]) is an 18-item self-report questionnaire investigating symptoms of Obsessive-Compulsive Disorder on a five-point Likert scale. The questionnaire consists of six subscales: “Washing”, “Ordering”, “Hoarding”, “Mental Neutralizing”, “Obsessing”, and “Checking”. The Italian version shows good psychometric properties that are comparable to the original one.

The current study highlighted a good internal consistency for all subscales and total scores (Table 2).

The Multidimensional Perfectionism Scale (MPS [58, 59]; the Italian version by [60]) is a 35-item self-report questionnaire investigating different perfectionistic traits on a five-point Likert scale. The MPS consists of six subscales: “Concern over Mistakes”, “Personal Standards”, “Parental Expectations”, “Parental Criticism”, “Doubting of Actions”, and “Organization”. The internal consistency was good (0.76 < Cronbach’s α < 0.93), and the concurrent validity was acceptable both in the original and in the Italian version. In the current study, all subscales showed good internal consistency, except for the “Parental Criticism” subscale in the Obesity/BED group, where Cronbach’s α was acceptable (Table 2).

The Beck Anxiety Inventory (BAI [61]; the Italian version by [62]) is a 21 item self-report questionnaire investigating the severity of anxiety symptoms on a four-point Likert scale. The BAI displayed excellent internal consistency (Cronbach’s α = 0.92) and a good test-retest reliability over 1 week (*r* = 0.75). The Italian version showed good psychometric properties. In a sample of students, its internal consistency was good (Cronbach’s α = 0.89), and the test-retest reliability was acceptable. The current study showed excellent internal consistency for each group (Table 2).

The Beck Depression Inventory-Second Edition (BDI-II [63]; the Italian version by [64]) is a 21 item self-report questionnaire rated on a four-point Likert scale, used to assess the severity of depression symptoms. The BDI-II showed excellent internal consistency in a sample of university students (Cronbach’s α = 0.93) and a clinical sample (Cronbach’s α = 0.92). The test-retest reliability across a period of a week was good (*r* = 0.93), as well as convergent and divergent validity. The Italian version showed good internal consistency, respectively, considering a sample of university students, patients with depression, and a group of individuals extracted from the general population (0.80 < Cronbach’s α < 0.87). The test-retest reliability across a period of a month was good (*r* = 0.76). The current study showed an excellent internal consistency (Table 2).

### Data analysis

Analyses were conducted with SPSS Statistics-Version 25. Cronbach’s alpha was calculated for all scales and subscales of the self-report questionnaires for each group.

A Chi-squared index was used to investigate the differences between groups in gender. Multivariate ANOVA was used to explore the differences between groups for age, school years, BMI, and the questionnaires’ total scores and subscales. For MANOVAs, Fisher’s F and Partial Eta Squared were reported as effect sizes. A Bonferroni corrected post-hoc test was performed to check for group differences concerning the questionnaires’ total scores and subscales. A Linear Regression Multiple Analysis was performed to examine if BMI and the total score of OCI-R, MPS, and EDI-3 predicted the EHQ total score.

## Results

### Differences between groups

Groups differed in gender (χ^2^ = 82.06; *p* < 0.001). Females were more represented in the AN/BN and Control groups (98.9% and 95.7%). Groups also differed in age (F_(3)_ = 126.23; η^2^ = 0.54; *p* < 0.001). The Obesity/BED group was older than the other ones, and the Diet group was older than the AN/BN and Control groups. Control group showed a higher level of school years than the other groups (F_(3)_ = 18.67; η^2^ = 0.15; *p* < 0.001), (F_(3)_ = 194.55; η^2^ = 0.65; *p* < 0.001). The Obesity/BED group had the highest BMI, while the AN/BN group had the lower one (F_(3)_ = 194.55; η^2^ = 0.65; *p* < 0.001) (Table 1).

### Differences between groups in Orthorexic features

Significant differences between groups were highlighted regarding the EHQ total score (F_(3)_ = 50.19; η^2^ = 0.32; *p* < .001), “Problems” (F_(3)_ = 80.00; η^2^ = 0.42; *p* < .001), “Knowledge” (F_(3)_ = 30.44, η^2^ = 0.22, *p* < .001) and “Feelings” (F_(3)_ = 17.49; η^2^ = 0.14; *p* < .001) subscales.

﻿For the EHQ-21 total score, post-hoc Bonferroni tests highlighted that AN/BN (M = 55.27; SD = 15.45) had higher score than Obesity/BED (M = 47.91; SD = 10.96) and Control (M = 36.22; SD = 7.54). Obesity/BED and Diet (M = 50.99; SD = 8.84) had higher scores than Control. The Diet group did not differ from clinical groups and scored higher than the Control.

Regarding the “Problems” subscale, AN/BN (M = 30.92; SD = 10.23) scored higher than Obesity/BED (M = 22.55; SD = 6.01), Diet (M = 22.69; SD = 5.86) and Control (M = 15.26; SD = 3.50) groups. Obesity/BED and Diet groups scored higher than the Control one.

In the “Feelings” subscale, AN/BN (M = 11.01; SD = 3.08), Obesity/BED (M = 11.42; SD = 2.82) and Diet (M = 11.91; SD = 2.52) groups scored higher than the Control (M = 9.11, SD =2.70), so that the Diet group score did not differ from that of the clinical groups.

Regarding the “Knowledge” subscale, Bonferroni post-hoc tests highlighted that the Diet group (M = 16.38; SD = 2.61) scored higher than AN/BN (M = 13.33; SD = 3.90), Obesity/BED (M = 13.94; SD = 3.55) and Control (M = 11.86; SD = 3.03) groups. AN/BN and Obesity/BED scored higher than the Control one (Table 2).

### Differences between groups in EDs symptoms

It was highlighted statistically significant difference between groups in the EDI-3 total score (F_(3)_ = 143.64; η^2^ = 0.57; *p* < .001) and along with other (Supplementary Material) in the “Drive for Thinness” (F_(3)_ = 119.72; η^2^ = 0.53; *p* < .001), “Bulimia” (F_(3)_ = 31.52; η^2^ = 0.23; *p* < .001), “Body Dissatisfaction” (F_(3)_ = 61.00, η^2^ = 0.36; *p* < .001). Regarding the EDI-3 total score and “Drive for Thinness”, AN/BN obtained higher rates than Obesity/BED, Diet, and Control. Obesity/BED scored higher than Diet and Control groups (Table 2). These results highlighted that the Diet group showed statistically significant lower scores than the clinical groups, not differing from the Control one. Regarding “Bulimia” and “Body Dissatisfaction” subscales, AN/BN scored higher than Diet and Control. In contrast, Obesity/BED scored higher than Diet and Control, highlighting that the Diet group showed statistically significantly lower scores than the clinical groups, not differing from the Control one.

### Differences between groups in perfectionism

Regarding the MPS total, there were significant differences between groups (F_(3)_ = 15.27; η^2^ = 0.12; *p* < .001). AN/BN scored statistically significantly higher than other groups in the MPS total score and some other subscales (Supplementary Material).

### Differences between groups in obsessive-compulsive symptoms, anxiety, and depression

It was highlighted a statistically significant difference between groups in the OCI-R total score (F_(3)_ = 30.96; η^2^ = 0.22; *p* < .001) and in other subscales (Supplementary Material). AN/BN (M = 25.77; SD = 14.96) scored higher than Obesity/BED (M = 13.53; SD = 13.74), Diet (M = 13.55; SD = 11.87), and Control (M = 8.55; SD = 9.52). The Diet group scored similarly to the Obesity/BED but higher than the Control (Table 2).

It was highlighted a statistically significant difference between groups in the BAI total score (F_(3)_ = 60.50; η^2^ = 0.36; *p* < .001). Post-hoc Bonferroni test highlighted that AN/BN scored higher (M = 26.67; SD = 14.46) than Obesity/BED (M = 9.36; SD = 10.45), Diet (M = 7.36; SD = 9.62) and Control (M = 9.50; SD = 7.80). These results underlined a similarity between the Diet group scores and those of the Obesity/BED and Control groups.

Regarding the BDI-II total score, there were statistically significant differences between groups (F_(3)_ = 97.75; η^2^ = 0.48; *p* < .001). In BDI-II total score, AN/BN (M = 30.14; DS = 14.49) scored significantly higher than the other three groups, and the Obesity/BED group scored higher than Diet (Table 2).

### Linear regression analysis

A Linear Regression Analysis was conducted for each group to investigate if BMI, obsessive-compulsive, perfectionistic, and eating disorder features predicted ON.

Regards the Diet group, the MPS total score (β = .36; *p* < .01) is a significant predictor of ON features (*R*^*2*^ = .238; F_(4,84)_ = 6.572). For the Obesity/BED group, both the MPS (β = .53; *p* < .01) and EDI-3 total scores (β = −.37; *p* < .05) have a predictive role (*R*^*2*^ = .21; F_(4,48)_ = 3.24); instead, the EDI-3 total score (β = .42; *p* < .001) and BMI (β = −.36; *p* < 0.001) predicted ON for the AN/BN group (*R*^*2*^ = .44; F_(4,85)_ = 16.51) (Table 3).

**Table 3:** Linear regression models

	F_(d.f.1, d.f.2)_	R^2^	β BMI (p)	β OCI-R-TOT (p)	β MPS-TOT (p)	β EDI-3-TOT (p)
**AN/BN**	16.51_(4,85)_	.44	−.36 (<.001)	.06 (n.s.)	.18 (n.s.)	.42 (<.001)
**Obesity/BED**	3.24_(4,48)_	.21	−.01 (n.s.)	.08 (n.s.)	.53 (<.01)	−.37 (<.05)
**DIET**	6.57_(4,84)_	.24	−.12 (n.s.)	.10 (n.s.)	.36 (<.01)	.06 (n.s.)

*AN/BN* Anorexia Nervosa/Bulimia Nervosa, *Obesity/BED* Obesity/Binge Eating Disorder, *n.s.* Not statistically significant, *BMI* Body Mass Index, *OCI-R* Obsessive Compulsive Inventory-*Revised*, *MPS* Multidimensional Perfectionism Scale, *EDI-3* Eating Disorder Inventory-3, *d.f.* degrees of freedom

## Discussion

The principal aim of this study was to investigate ON characteristics, considering differences between clinical samples at risk of showing greater ON tendencies. Specifically, groups consisted of patients diagnosed with AN/BN, obesity, and people who voluntarily followed a diet. These groups were compared with a Control one, who was not following any diet.

Results highlighted that clinical and Diet groups displayed higher orthorexic tendencies than the Control one in the EHQ total score and subscales. Moreover, AN/BN and Diet groups did not differ in the total score of the EHQ and showed higher scores than Obesity/BED and Control. These results confirm that individuals who voluntarily follow a diet and those diagnosed with an ED share similarities in ON characteristics [13, 14, 24].

Regarding the EHQ “Problems” subscale, the Obesity/BED group scores did not differ from the Diet group, but they are higher than controls. In line with previous evidence [19], AN/BN showed significantly higher scores than other groups in this subscale. At the time of administration, AN/BN patients were admitted to clinics for psycho-nutritional rehabilitation. As reported by health care professionals, during hospitalization, patients were invited to reflect on their previous dysfunctional eating habits as a part of the treatment. In addition to the hospitalization factor, the highest score obtained by the AN/BN could be associated with the severity of their disease: unlike the other groups, the AN/BN reached the clinical cut-off in the questionnaires used to investigate anxiety (44%), depressive (53.30%) and typical OCD symptoms (52.20%).

In the EHQ “Knowledge” subscale, scores obtained by the AN/BN did not differ from those obtained by the Obesity/BED; however, the Diet group showed scores significantly higher compared to clinical groups. People who voluntarily follow a diet could believe that they have more information about healthy eating and that their dieting behaviors could be necessary.

There were no significant differences between the three experimental groups on the EHQ “Feelings” subscale. Nevertheless, these groups showed significantly higher scores than the Control one, probably because following a diet could experience positive and satisfying emotions in exercising control on their healthy eating [65]. These results confirm that ON and EDs share similarities related to Problems, Knowledge, and Feeling in pursuing a healthy diet [10, 12, 19].

Specifically, lower BMI levels and higher scores at the EDI-3 are related to ON symptomatology in the AN/BN group, highlighting that the attempt to control weight and focalization on body shape could be related to higher orthorexic tendencies. Furthermore, during recovery, patients with AN/BN learn to follow a structured diet as part of their treatment, increasing ON scores.

The Obesity/BED group had greater ON behaviors than the Control one but lower than the AN/BN group. Moreover, it showed significantly more depressive symptoms than the Diet and Control groups, although the score remains below the clinical cut-off. Even though the scores on the clinical scales which investigated the symptomatology of EDs are significantly higher than the Diet and the Control, the Obesity/BED group did not show the same severity of psychopathology as patients with AN/BN. Only in the EDI-3 “Bulimia” and “Body Dissatisfaction” subscales AN/BN and Obesity/BED groups did not differ. To better understand characteristics that could be related to ON features, a linear regression analysis was performed. In the Obesity/BED group, perfectionistic traits and EDs symptomatology respectively predict ON directly and inversely. As opposed to what had been expected, in the AN/BN group, the EDI-3 total score directly predicted the EHQ total score, while in the Obesity/BED group, a greater presence of EDs symptoms predisposed to lower ON tendencies. Thus, in the Obesity/BED group, EDs total score did not represent a factor related to ON. However, future studies should point out that the “Bulimia” and the “Body Dissatisfaction” EDI-3 subscales could be characteristics that better explain orthorexic tendencies than the EDI-3 total score. In this group, the MPS total score directly predicts orthorexic tendencies. Perfectionism could predispose to strict adherence to healthy eating habits adopted to improve health, reflected in an increase in ON scores. People with obesity are at risk of developing precarious health conditions or have already developed them [66], and an overconcern about these conditions could be related to ON characteristics.

In the Diet group, the MPS total score predicted the EHQ total score. Therefore, a rigid adherence to healthy eating habits and a condescending attitude toward healthy diet-related aspects could interact with perfectionistic traits. In this group, the typical AN/BN symptomatology was not present, and in no questionnaire assessing psychopathological characteristics the clinical cut-off has been reached. When perfectionistic traits are shown, a voluntary diet could predispose to greater ON features.

The literature highlighted that ON and OCD have similar characteristics [8–10]. Nevertheless, in the current study, OCD symptomatology is not related to ON tendencies in no group, even if in the AN/BN group, the clinical cut-off for obsessive-compulsive symptoms had been reached; this is in line with previous results showing that ON does not seem to be related to OCD [10, 67].

Individuals who pursue a diet share similarities with those who have an eating disorder related to ON positive emotions, behaviors, and problems. Moreover, perfectionistic traits seem to predispose to higher ON characteristics. In general, these results confirm the ON as an aspect of the main eating disorders category.

### Limitations and future directions

One of the limitations of this study was that groups differ in sociodemographic characteristics such as gender, age, and education, even if literature put in evidence heterogeneous results regarding these aspects [12, 68].

Another limitation was that the Diet group was recruited by a single dietician who prescribed an unconventional diet. In this respect, future research should compare orthorexic tendencies in samples of subjects following conventional and unconventional diets. The current study could be replicated in other at-risk samples, such as athletes, dancers, or gymnasts who follow a diet, to understand the role of perfectionism on orthorexic tendencies in these populations.

Another limit concerns the lack of data about ON characteristics over time; for this purpose, ON could be further investigated in EDs samples to understand whether it is a prodromal or post-treatment condition of EDs.

Further studies should investigate the relationship between ON, EDs, OCD, and health anxiety features to assess similarities/differences of these disorders regarding content, process, and functioning. Finally, future research should also investigate, in addition to perfectionism, other personality traits that may predispose ON tendencies.

## Conclusion

In this study, both people who adopt a “non-conventional” diet and those who have received an eating disorder diagnosis and follow a diet within hospital treatment show orthorexic tendencies. Therefore, pursuing a diet could represent a prodrome of an EDs or an interfering behavior within the treatment. In clinical practice and the EDs assessment, it would be helpful to consider also orthorexic aspects. Moreover, studies on this subject should suggest to health care professionals to consider possible problems related to ON in their patients on a diet.

## Supplementary Information

**Additional file 1.**

## Data Availability

The datasets used and/or analyzed for the present study will be provide by the corresponding author if any reasonable request would be raised.
