# Supplementary material for: Orthorexia Nervosa: differences between clinical and non-clinical samples
Source: BMC Psychiatry. 2021 Jul 8;21:341. doi: 10.1186/s12888-021-03348-2 (PMC8265101; doi:10.1186/s12888-021-03348-2)
Supplement: Supplementary file 1 — Additional file 1. [file 12888_2021_3348_MOESM1_ESM.docx]

| **Supplementary Material.**  Comparisons between the AN/BN, Obesity/BED, Diet and Control groups | | | | | | | | | |
| --- | --- | --- | --- | --- | --- | --- | --- | --- | --- |
|  | **AN/BN (1)**  **N=90** | **Obesity/BED (2)**  **N=54** | **DIET (3)**  **N=91** | **CONTROL (4)**  **N=94** | **F _(d.f.)_** | ***p*** | **Partial η^2^** | **Bonferroni post-hoc comparison** **(p)** | **α** |
| **EHQ-21-TOT**  **MEAN (SD)** | 55.27 (15.45) | 47.91  (10.96) | 50.99  (8.84) | 36.22  (7.54) | 50.19_(3)_ | <.001 | .32 | **1>2;4 (<.001)**  **4<2;3 (<.001)** | (1)=.93  (2)=.88  (3)=.85  (4)=.85 |
| **EHQ-21-PROBLEMS MEAN (SD)** | 30.92  (10.23) | 22.55  (6.01) | 22.69  (5.86) | 15.26  (3.50) | 80.00_(3)_ | <.001 | .42 | **1>2;3;4 (<.001)**  **4<2;3 (<.001)** | (1)=.92  (2)=.75  (3)=.81  (4)=.75 |
| **EHQ-21-KNOWLEDGE MEAN (SD)** | 13.33  (3.90) | 13.94  (3.55) | 16.38  (2.61) | 11.86  (3.03) | 30.44_(3)_ | <.001 | .22 | **4<1;2 (<.05)**  **3>1;2;4 (<.001)** | (1)=.79  (2)=.75  (3)=.74  (4)=.79 |
| **EHQ-21-FEELINGS MEAN (SD)** | 11.01 (3.08) | 11.42  (2.82) | 11.91  (2.52) | 9.11  (2.70) | 17.49_(3)_ | <.001 | .14 | **4<1;2;3 (<.001)** | (1)=.61  (2)=.72  (3)=.65  (4)=.71 |
| **EDI-3-TOT MEAN (SD)** | 179.84  (55.15) | 121.89  (60.53) | 57.97  (30.62) | 62.18  (36.62) | 143.64_(3)_ | <.001 | .57 | **1>2;3;4 (<.001)**  **2>3;4 (<.001)** | (1)=.96  (2)=.97  (3)=.94  (4)=.96 |
| **EDI-3-DRIVE FOR THINNESS MEAN (SD)** | 17.99  (7.34) | 12.98  (6.95) | 5.92  (3.25) | 4.12  (4.21) | 119.72_(3)_ | <.001 | .53 | **1>2;3;4 (<.001)**  **2>3;4 (<.001)** | (1)=.85  (2)=.79  (3)=.72 (4)=.82 |
| **EDI-3-BULIMIA MEAN (SD)** | 10.28  (10.17) | 9.57  (8.14) | 2.65  (3.56) | 2.86  (3.20) | 31.52_(3)_ | <.001 | .23 | **1>3;4 (<.001)**  **2>3;4 (<.001)** | (1)=.93  (2)=.90  (3)=.84  (4)=.77 |
| **EDI-3-BODY DISSATISFACTION MEAN (SD)** | 25.93  (9.94) | 24.08  (9.34) | 10.73  (8.42) | 12.18  (9.15) | 61.00_(3)_ | <.001 | .36 | **1>3;4 (<.001)**  **2>3;4 (<.001)** | (1)=.87  (2)=.81  (3)=.87  (4)=.91 |
| **EDI-3-LOW SELF ESTEEM MEAN (SD)** | 15.52  (6.51) | 7.21  (6.34) | 3.70  (4.22) | 4.46  (4.31) | 93.61_(3)_ | <.001 | .46 | **1>2;3;4 (<.001)**  **2>3;4 (<.05)** | (1)=.90  (2)=.86  (3)=.88  (4)=.87 |
| **EDI-3-PERSONAL ALIENATION MEAN (SD)** | 14.82  (6.04) | 8.17  (6.71) | 3.90  (3.97) | 4.18  (3.82) | 91.12_(3)_ | <.001 | .46 | **1>2;3;4 (<.001)**  **2>3;4 (<.001)** | (1)=.74  (2)=.84  (3)=.76  (4)=.79 |
| **EDI-3-INTERPERSONAL INSECURITY MEAN (SD)** | 13.11  (6.30) | 8.13  (6.10) | 6.00  (4.60) | 6.11  (5.55) | 31.86_(3)_ | <.001 | .23 | **1>2;3;4 (<.001)** | (1)=.83  (2)=.79  (3)=.81  (4)=.88 |
| **EDI-3-INTERPERSONAL ALIENATION MEAN (SD)** | 13.21  (5.79) | 8.26  (5.26) | 5.81  (3.65) | 5.56  (3.99) | 52.11_(3)_ | <.001 | .33 | **1>2;3;4 (<.001)**  **2>3;4 (<.05)** | (1)=.79  (2)=.77  (3)=.70  (4)=.79 |
| **EDI-3-INTEROCEPTIVE DEFICITS MEAN (SD)** | 19.57  (8.56) | 10.43  (8.11) | 3.53  (3.88) | 3.78  (4.43) | 127.11_(3)_ | <.001 | .54 | **1>2;3;4 (<.001)**  **2>3;4 (<.001)** | (1)=.87  (2)=.86  (3)=.81  (4)=.86 |
| **EDI-3-EMOTIONAL DYSREGULATION MEAN (SD)** | 10.24  (6.19) | 5.81  (5.33) | 2.32  (2.56) | 3.13  (3.32) | 57.55_(3)_ | <.001 | .35 | **1>2;3;4 (<.01)**  **2>3;4 (<.01)** | (1)=.78  (2)=.71  (3)=.75  (4)=.75 |
| **EDI-3-PERFECTIONISM MEAN (SD)** | 10.47  (5.02) | 7.85  (5.07) | 4.02  (2.67) | 4.51  (2.84) | 53.11_(3)_ | <.001 | .33 | **1>2;3;4 (<.001)**  **2>3;4 (<.001)** | (1)=.70  (2)=.70  (3)=.65  (4)=.59 |
| **EDI-3-MATURITY FEARS MEAN (SD)** | 14.26  (7.85) | 11.25  (7.10) | 6.34  (4.15) | 7.65  (4.93) | 30.99_(3)_ | <.001 | .22 | **1>2;3;4 (<.05)**  **2>3;4 (<.01)** | (1)=.85  (2)=.76  (3)=.63  (4)=.80 |
| **OCI-R-TOT MEAN (SD)** | 25.77  (14.96) | 13.53  (13.74) | 13.55  (11.87) | 8.55  (9.52) | 30.96_(3)_ | <.001 | .22 | **1>2;3;4 (<.001)**  **3>4 (<.05)** | (1)=.90  (2)=.94  (3)=.91  (4)=.91 |
| **OCI-R-WASHING MEAN (SD)** | 2.98  (3.24) | 1.34  (2.29) | 1.46  (2.12) | 1.00  (1.87) | 11.44_(3)_ | <.001 | .10 | **1>2;3;4 (<.001)** | (1)=.73  (2)=.73  (3)=.70  (4)=.75 |
| **OCI-R-CHECKING**  **MEAN (SD)** | 3.52  (3.23) | 2.09  (2.52) | 2.54  (2.91) | 1.13  (1.85) | 12.52_(3)_ | <.001 | .10 | **1>2;4 (<.05)**  **3>4 (<.01)** | (1)=.79  (2)=.83  (3)=.78  (4)=.70 |
| **OCI-R-ORDERING**  **MEAN (SD)** | 5.50  (4.04) | 2.64  (2.81) | 3.02  (2.79) | 2.10  (2.62) | 20.26_(3)_ | <.001 | .16 | **1>2;3;4 (<.001)** | (1)=.90  (2)=.83  (3)=.82  (4)=.87 |
| **OCI-R-OBSESSING**  **MEAN (SD)** | 6.90  (3.55) | 2.49  (3.20) | 2.03  (2.60) | 1.74  (2.42) | 60.56_(3)_ | <.001 | .36 | **1>2;3;4 (<.001)** | (1)=.78  (2)=.85  (3)=.75  (4)=.89 |
| **OCI-R-HOARDING**  **MEAN (SD)** | 4.16  (3.20) | 3.60  (3.40) | 3.14  (2.87) | 1.99  (2.43) | 8.98_(3)_ | <.001 | .08 | **4<1;2;3 (<.05)** | (1)=.74  (2)=.85  (3)=.76  (4)=.76 |
| **OCI-R-MENTAL NEUTRALIZING MEAN (SD)** | 2.71  (3.39) | 1.36  (2.33) | 1.35  (2.00) | 0.60  (1.90) | 11.44_(3)_ | <.001 | .10 | **1>2;3;4 (<.05)** | (1)=.76  (2)=.78  (3)=.76  (4)=.92 |
| **MPS-TOT**  **MEAN (SD)** | 113.94  (23.73) | 93.32  (22.61) | 94.43  (24.99) | 95.53  (24.18) | 15.27_(3)_ | <.001 | .12 | **1>2;3;4 (<.001)** | (1)=.94  (2)=.92  (3)=.95  (4)=.95 |
| **MPS-PERSONAL STANDARD**  **MEAN (SD)** | 23.56  (6.33) | 19.45  (5.17) | 21.13  (6.73) | 21.20  (6.32) | 5.28_(3)_ | <.01 | .05 | **1>2 (<.01)** | (1)=.83  (2)=.75  (3)=.88  (4)=.89 |
| **MPS-CONCERN OVER MISTAKES**  **MEAN (SD)** | 31.72  (8.72) | 23.04  (8.87) | 21.70  (8.71) | 22.09  (8.04) | 27.55_(3)_ | <.001 | .20 | **1>2;3;4 (<.001)** | (1)=.92  (2)=.91  (3)=.92  (4)=.92 |
| **MPS-DOUBTING OF ACTION MEAN (SD)** | 13.43  (4.07) | 9.96  (3.76) | 9.27  (3.85) | 9.71  (3.80) | 21.61_(3)_ | <.001 | .17 | **1>2;3;4 (<.001)** | (1)=.83  (2)=.75  (3)=.79  (4)=.83 |
| **MPS-PARENTAL EXPECTATION MEAN (SD)** | 11.21  (5.19) | 10.94  (5.39) | 10.58  (5.41) | 10.47  (4.79) | 0.38_(3)_ | *n.s.* | .004 | *n.s.* | (1)=.89  (2)=.89  (3)=.92  (4)=.89 |
| **MPS-PARENTAL CRITICISM MEAN (SD)** | 10.60  (4.59) | 9.23  (3.59) | 8.64  (3.76) | 8.00  (3.51) | 7.33_(3)_ | <.001 | .06 | **1>3;4 (<.01)** | (1)=.82  (2)=.61  (3)=.75  (4)=.77 |
| **MPS-ORGANIZATION MEAN (SD)** | 23.42  (5.76) | 20.70  (6.23) | 23.10  (4.66) | 22.06  (4.93) | 3.52_(3)_ | <.02 | .03 | **1>2 (<.05)** | (1)=.91  (2)=.93  (3)=.90  (4)=.92 |
| **BAI-TOT MEAN (SD)** | 26.67 (14.46) | 9.36  (10.45) | 7.36  (9.62) | 9.50  (7.80) | 60.50_(3)_ | <.001 | .36 | **1>2;3;4 (<.001)** | (1)=.93  (2)=.93  (3)=.94  (4)=.88 |
| **BDI-II-TOT MEAN (SD)** | 30.14  (14.49) | 11.87  (11.44) | 6.88  (7.49) | 7.10  (7.81) | 97.75_(3)_ | <.001 | .48 | **1>2;3;4 (<.001)**  **2>3 (<.05)** | (1)=.93  (2)=.94  (3)=.90  (4)=.92 |

**Notes.** BMI= Body Mass Index; EHQ-21= Eating Habits Questionnaire-21; BAI = Beck Anxiety Inventory; BDI-II: Beck Depression Inventory-II OCI-R = Obsessive Compulsive Inventory-*Revised;* MPS = Multidimensional Perfectionism Scale; EDI-3 = Eating Disorder Inventory-3; AN/BN = AnorexiaNervosa/BulimiaNervosa; Obesity/BED = Obesity/Binge Eating Disorder; SD = Standard Deviation; *n.s.* = Not statistically significant; d.f. = degrees of freedom; α = Cronbach’s Alpha coefficient.
